# Vitamin D and skeletal health in autoimmune bullous skin diseases: a case control study

**DOI:** 10.1186/s13023-015-0230-0

**Published:** 2015-02-03

**Authors:** Angelo Valerio Marzano, Valentina Trevisan, Elisa Cairoli, Cristina Eller-Vainicher, Valentina Morelli, Anna Spada, Carlo Crosti, Iacopo Chiodini

**Affiliations:** Dipartimento di Fisiopatologia Medico-Chirurgica e dei Trapianti, University of Milan, Unit of Dermatology, Fondazione IRCCS Ca’ Granda-Ospedale Maggiore Policlinico, Milan, Italy; Department of Clinical Sciences and Community Health, University of Milan, Milan, Italy; Unit of Endocrinology and Metabolic Diseases, Fondazione IRCCS Cà Granda-Ospedale Maggiore Policlinico, Milan, Italy

**Keywords:** Vitamin D, Autoimmune bullous skin diseases, Vertebral fractures

## Abstract

**Background:**

The presence of hypovitaminosis D in patients with autoimmune bullous skin diseases, such as pemphigus vulgaris (PV) and bullous pemphigoid (BP), is debated. In a previous study we found an increased prevalence of vertebral fractures (VFx) and hypovitaminosis D in PV and BP patients. The present study extends the sample size of the previous one, for investigating the 25-hydroxyvitamin D (25OHVitD) levels in relation with the skeletal health and disease intensity in these patients.

**Methods:**

The previous study was performed in 13 PV and 15 BP patients and 28 controls. Data from 39 additional patients (22 PV and 17 BP) were now added. Eventually, we studied 67 patients (35 PV, 32 BP, 51 females), aged 64.7 ± 16.9 years and 67 age- gender- and body mass index-matched controls. In all subjects, serum 25OHVitD, calcium and alkaline phosphatase (ALP) levels were measured, bone mineral density (BMD) was evaluated by Dual-energy X-ray. Absorptiometry at lumbar spine (LS) and femoral neck (FN) and the presence of VFx were ascertained by visual assessment from spinal radiographs. In patients, the disease intensity was evaluated by the autoimmune bullous skin disorder intensity score (ABSIS).

**Results:**

As compared with controls, both PV and BP patients showed lower 25OHVitD (22.2 ± 11.1 vs 13.9 ± 8.3 ng/mL, p < 0.001 and 22.4 ± 14.9 vs 9.5 ± 7.7 ng/mL, p < 0.0001, respectively) and higher prevalence of severe hypovitaminosis D (22.9 vs 48.6%, p < 0.02 and 31.1 vs 75.0%, p < 0.0001, respectively) and VFx (28.6 vs 57.1%, p = 0.03 and 34.4 vs 62.5%, P = 0.02, respectively). In both PV and BP patients, LS and FN BMD did not differ from controls. In the whole patients’ group, ABSIS score was inversely associated with 25OHVitD levels (R = −0.36, p < 0.005), regardless of age (β = −3.2, P = 0.009).

**Conclusions:**

PV and BP patients have an increased prevalence of hypovitaminosis D and VFx. The extended study shows, for the first time, that the 25OHVitD levels are inversely associated with disease intensity and that VFx occur in spite of a not reduced BMD.

**Electronic supplementary material:**

The online version of this article (doi:10.1186/s13023-015-0230-0) contains supplementary material, which is available to authorized users.

## Background

Recently, vitamin D has been suggested to influence the immune system, triggering or exacerbating autoimmunity [1], and hypovitaminosis D has been shown to be associated with the prevalence and severity of different autoimmune disorders [2-4].

Pemphigus vulgaris (PV) and bullous pemphigoid (BP) are rare (incidence 0.75-5 and 6–7 cases per million per year, respectively) and potentially life-threatening autoimmune bullous skin disorders [5-9]. PV is caused by autoantibodies to the desmosomal desmogleins (Dsg3-specific autoantibodies and Dsg1-specific autoantibodies), while two hemidesmosomal antigens, BP180 and BP230, are involved in BP [10]. To date, immunosuppressive agents, particularly corticosteroids, represent the treatment of choice for both diseases [11,12]. However, regardless of the use of corticosteroid, autoimmune conditions may lead to a skeletal damage characterized by decreased bone mineral density (BMD) and increased risk of fractures [13]. The skeletal involvement in autoimmune diseases is thought to be related to the effect on bone cells of several factors, notably co-stimulatory receptors and cytokines [13], which may be regulated by the 25-hydroxyvitamin D (25OHVitD) levels [14].

Recently, the issue of hypovitaminosis D in autoimmune bullous skin disorders has become a matter of debate, since some studies suggested an increased prevalence of hypovitaminosis D in PV and BP patients [15,16], while others failed to find such associations [17,18]. These discordances may be related to the small sample size of the available studies and to the frequency of hypovitaminosis D even in the healthy populations [19]. However, this topic is of pathophysiologic importance since the condition of hypovitaminosis D has been suggested to play a role in the pathogenesis of a number of immune-mediated inflammatory disorders, including autoimmune bullous diseases [16,17,20]. On the other hand, from a clinical point of view, the presence of a bone damaging cytokine milieau together with a condition of hypovitaminosis D may render the PV and BP patients at risk of fragility fractures [21].

Therefore, the aim of the present study was to evaluate in a large sample of PV and BP patients, the skeletal metabolism, 25OHVitD levels, bone mineral density (BMD) and the prevalence and severity of vertebral fractures (VFx) in relation to the disease intensity.

## Methods

### Patients

The present investigation is the extension of a case–control study, performed in our Hospital between June 2009 and June 2011, in which 13 PV and 15 BP Caucasian patients and 28 matched controls were included [15]. Between July 2012 and June 2014, 49 additional Caucasian patients referred to our Dermatology Unit for PV (n = 28) or BP (n = 21) were consecutively evaluated for the study inclusion. Among these, 10 patients (6 with PV and 4 with BP) were excluded for the presence of already known concomitant active diseases (endocrine, neoplastic or inflammatory) and/or intake of drugs (i.e. bone anabolic or antiresorptive drugs) potentially affecting bone metabolism and mass. Eventually, 39 patients (22 with PV and 17 with BP) were enrolled in the extension study.

As a prerequisite for the enrolment in the study, the detection of autoantibodies in the skin and in serum had to be positive in all patients [22]. In all PV patients, the diagnosis of active disease was based on typical blisters and/or erosions of the skin and mucous membranes, together with the presence of intercellular IgG deposition with or without C3 on direct immunofluorescence (DIF) and detection of circulating Dsg3-specific autoantibodies (with or without Dsg1-specific autoantibodies) by enzyme-linked immunosorbent assay (ELISA) [11].

In all BP patients, the diagnosis of active disease was established on the basis of the presence of blisters and/or erosions of the skin and/or erythematous-oedematous skin lesions as well as the typical immunopathological criteria [23], such as linear deposition of IgG and/or C3 at the basement membrane zone (BMZ) on DIF and circulating levels of anti-BP180 and/or anti-BP230 autoantibodies on ELISA. At the enrolment no BP patients had mucosal involvement.

At the enrolment all PV and BP patients showed active disease and were “baseline”. Baseline is defined as the day that therapy is started by a physician, according to recently published recommendations by an international panel of experts [24]. In all PV and BP patients, the disease intensity was assessed by the autoimmune bullous skin disorder intensity score (ABSIS) which is based on two clinical criteria, such as the extent of the affected area and the quality of the skin and mucosal lesions [25]. We decided to evaluate only cutaneous ABSIS score for a reason of homogeneity, since BP patients presented only with cutaneous lesions.

On the basis of the same exclusion criteria used for PV and BP patients, in the extension study, we enrolled as control group 39 Caucasian subjects selected from the cohort of inpatients affected with an incidentally discovered adrenal mass (adrenal incidentaloma, AI) and admitted in the Endocrinology Unit of our hospital for the hormonal evaluations. The AI diagnosis was based on the finding of unilateral adrenal mass by non-invasive imaging methods of the abdomen, performed for unrelated diseases. At computed tomography, all adrenal masses showed typical features of adrenocortical adenomas and all control subjects did not show clinical and/or biochemical signs of subclinical hypercortisolism, pheochromocytoma and aldosteronoma. Among the AI subjects, 35 and 32 were selected and matched for gender, age and body mass index (BMI) with PV and BP patients, respectively, and were evaluated in the same month of the year of his/her respective PV or BP patient.

We decided to enroll patients with adrenocortical adenomas in order to avoid the possible bias related to a different setting (i.e. inpatient vs outpatient). Indeed, as for PV and BP patients, the subjects bearing an adrenal mass were admitted as inpatients for the complex endocrine workup. Eventually, only patients without adrenal hyperfunction were enrolled in the study and served as controls.

In control subjects, the skin type was comparable to their respective PV and BP patients as evaluated by the Fitzpatrick Scale (PV Group: 11 patients with skin type II, 18 with skin type III and 6 with skin type IV; PV Controls: 9 subjects with skin type II, 21 with skin type III and 5 with skin type IV; BP Group: 16 patients with skin type II, 13 with skin type III and 3 with skin type IV; BP Controls: 18 subjects with skin type II, 11 with skin type III and 3 with skin type IV).

The protocol was approved by our Institutional Review Board (Ethical Committee Fondazione IRCCS Cà Granda Milan, Italy) and all the subjects gave their written informed consent before participating in the study.

We present data regarding patients enrolled in the extension study and we provide a pooled analysis of patients and controls enrolled in the previous and extension study.

### Methods

At the study entry, in all subjects serum 25-hydroxyvitamin D (25OHVitD), serum calcium corrected for serum albumin (reference interval 8.4-10.2 mg/dL), and total alkaline phosphatase (ALP) (reference interval 35–104 UI/L) were measured by standard colorimetric techniques. Serum 25OHVitD levels were measured by radioimmunoassay (RIA; DiaSorin) (reference interval: 30–120 ng/ml) [14]. Severe hypovitaminosis D was defined as the presence of 25OHD levels below 12 ng/mL (reference interval: 30–120 ng/ml). During the sample collection visit, the daily sun exposure over the previous week was assessed via a dedicated questionnaire [15]. Bone mineral density (BMD) was measured by Dual-energy X-ray Absorptiometry (DXA) (Hologic Discovery, Watham MA, USA) at lumbar spine (LS) and femoral neck (FN) and expressed as standard deviation (SD) units in relation to the reference healthy population of same age (Z-score) and of the young adults (T-score). Diagnosis of osteoporosis was made in the presence of T-score at any site < −2.5 and/or presence of clinical or asymptomatic low energy fractures. Vertebral fractures were diagnosed on visual inspection of conventional spinal radiographs in lateral and antero-posterior projection using the semi-quantitative visual assessment, as previously reported [15].

### Statistical analysis

Statistical analysis was performed by SPSS version 21.0 statistical package (SPSS Inc, Chicago, IL). The results are expressed as mean ± SD. Categorical variables were compared by *χ*2 test or Fischer exact test, as appropriate. For each variable, the normality of distribution was tested with the W statistic of Shapiro-Wilk. Normally distributed data were compared, after testing homogeneity of variance, by Student’s *t* test. The Mann–Whitney test was used when the normality test failed. The associations between variables were tested by Pearson’s product moment correlation or Spearman’s rank order correlation, as appropriate. The multivariate regression analysis was used to assess the independent association between ABSIS score, 25OHVitD levels and age, that were shown to be associated among themselves by Pearson’s product moment correlation or Spearman’s rank order correlation. P-values of less than 0.05 were considered significant.

## Results

The comparison of the clinical characteristics between the group of patients and their matched controls enrolled in the extension study is summarized in Additional file 1: Table S1. Patients showed lower 25OHVitD and ALP levels and higher prevalence of severe hypovitaminosis D and VFx than controls, while BMD at any site was comparable. Calcium levels tended to be lower in patients than in controls, while the Sun Exposure Score and the prevalence of current smokers and of patients with history of clinical fractures were comparable between patients and controls. In the control group mean vitamin D levels were in the range of insufficiency (i.e. between 20 and 30 ng/mL), while calcium and ALP levels were within the normal range.

The comparison of the clinical characteristics between the whole group of patients and their matched controls included in both previous and extension study is summarized in Additional file 1: Table S2. Beside age, gender and BMI, the Sun Exposure Score and the prevalence of current smokers and of patients with history of clinical fractures were comparable between patients and controls. Patients showed lower 25OHVitD, calcium and ALP levels and higher prevalence of severe hypovitaminosis D and VFx than controls, while BMD at any site was comparable.

The clinical characteristics between PV and BP patients and their matched controls are reported in Table 1. In PV and BP patients age, gender, BMI, Sun Exposure Score and the prevalence of subjects with history of clinical fractures and of current smokers were comparable as compared to the matched controls.

**Table 1:** **Characteristics of the whole sample of patients with Pemphigus Vulgaris (PV) and Bullous Pemphigoid (BP) and the respective controls**

	PV controls (n = 35)*	PV patients (n = 35)*	**P value**	BP controls (n = 32)*	BP patients (n = 32)*	**P value**
**Age (years)**	53.7 ± 14.9 (27 – 79)	55.5 ± 14.6 (27 – 79)	0.608	76.7 ± 9.2 (59 – 90)	79.0 ± 9.9 (48 – 90)	0.324
**Gender (females)**	19 (54.3)	19 (54.3)	1.000	17 (53.1)	17 (53.1)	1.000
**BMI (kg/m** ^**2**^ **)**	26.0 ± 4.3 (19.9 –37.0)	25.1 ± 5.0 (18.4 – 39.0)	0.661	27.9 ± 4.6 (20.3 – 36.0)	26.7 ± 4.0 (17.6 – 36.7)	0.284
**N. of current smokers (%)**	10 (28.6)	7 (20.0)	0.403	5 (15.6)	3 (9.4)	0.708
**N. of subjects with history of clinical fractures (%)**	4 (11.4)	1 (2.9)	0.356	5 (15.6)	3 (9.4)	0.708
**Sun exposure score**	13.7 ± 6.9 (4 – 28)	14.1 ± 5.8 (7 – 32)	0.791	9.9 ± 7.4 (2 – 26)	9.9 ± 7.3 (2 – 33)	0.851
**Calcium (mg/dL)**	9.2 ± 0.3 (8.7 – 9.7)	9.1 ± 0.4 (8.2 – 10.0)	0.513	9.3 ± 0.3 (8.6 – 10.0)	9.0 ± 0.3 (8.3 – 9.8)	0.001
**25(OH) Vitamin D (ng/mL)**	22.2 ± 11.1 (6.4 – 53.0)	13.9 ± 8.3 (4.0 – 34.8)	0.001	22.4 ± 14.9 (4.0 – 79.0)	9.5 ± 7.7 (4.0 – 31.8)	0.000
**N. of subjects with severe hypovitaminosis D (%)**	8 (22.9)	17 (48.6)	0.025	10 (31.3)	24 (75.0)	0.000
**Alkaline phosphatase (U/L)**	72.7 ± 19.1 (38 – 115)	60.4 ± 19.5 (35 – 128)	0.013	71.0 ± 17.2 (39 – 105)	64.9 ± 18.2 (34 – 105)	0.173
**LS BMD (Z-score)**	0.21 ± 1.42 (−2.0 – 3.7)	0.35 ± 1.67 (−3.2 – 3.3)	0.717	−0.03 ± 1.47 (−2.6 – 3.8)	0.06 ± 1.65 (−2.9 – 3.1)	0.831
**FN BMD (Z-score)**	−0.14 ± 1.04 (−1.6 – 2.7)	0.00 ± 1.31 (−2.9 – 3.0)	0.614	−0.26 ± 1.0 (−2.0 – 2.2)	−0.20 ± 0.83 (−1.7 – 1.8)	0.766
**N. of subjects with T-score < −2.5 at any site (%)**	4 (11.4)	8 (22.9)	0.342	9 (28.1)	12 (37.5)	0.424
**N. of subjects with vertebral fractures (%)**	10 (28.6)	20 (57.1)	0.029	11 (34.4)	20 (62.5)	0.024

Data are mean ± SD with range or percentage in parentheses for continuous or categorical variable, respectively.

*The table includes data from an already published study [reference #15] in which 28 patients (13 with PV and 15 with BP) and 28 matched controls were included, together with data from the extension arm of the study on 39 patients (22 with PV and 17 with BP) and 39 matched controls.

Severe hypovitaminosis D was defined in the presence of 25OHVitD levels <12 ng/mL (reference interval: 30–120 ng/ml).

Sun Exposure score: participants’ recollection of daily sun exposure over the previous week was assessed via a questionnaire administered (see reference #30)

Z-score, T-score: difference in standard deviation units in relation to the reference healthy population of same age (Z-score) and of the young adults (T-score).

Alkaline Phosphatase reference interval: 35–104 U/L. BMI: body mass index; BMD: bone mineral density; LS: lumbar spine; FT: total femur; FN: femoral neck.

As compared to controls, PV patients showed lower 25OHVitD and alkaline phosphatase levels and higher prevalence of severe hypovitaminosis D and VFx, while calcium levels and BMD at any site were comparable. No correlation was found between Dsg3-specific autoantibodies or Dsg1-specific autoantibodies and 25OHVitD levels.

Patients with BP showed lower calcium and 25OHVitD levels and higher prevalence of severe hypovitaminosis D and VFx than controls, while BMD at any site was comparable. In BP patients, 25OHVitD levels were inversely correlated with serum BP180 antibody levels (R = −0.40, p = 0.04).

**Figure 1:**
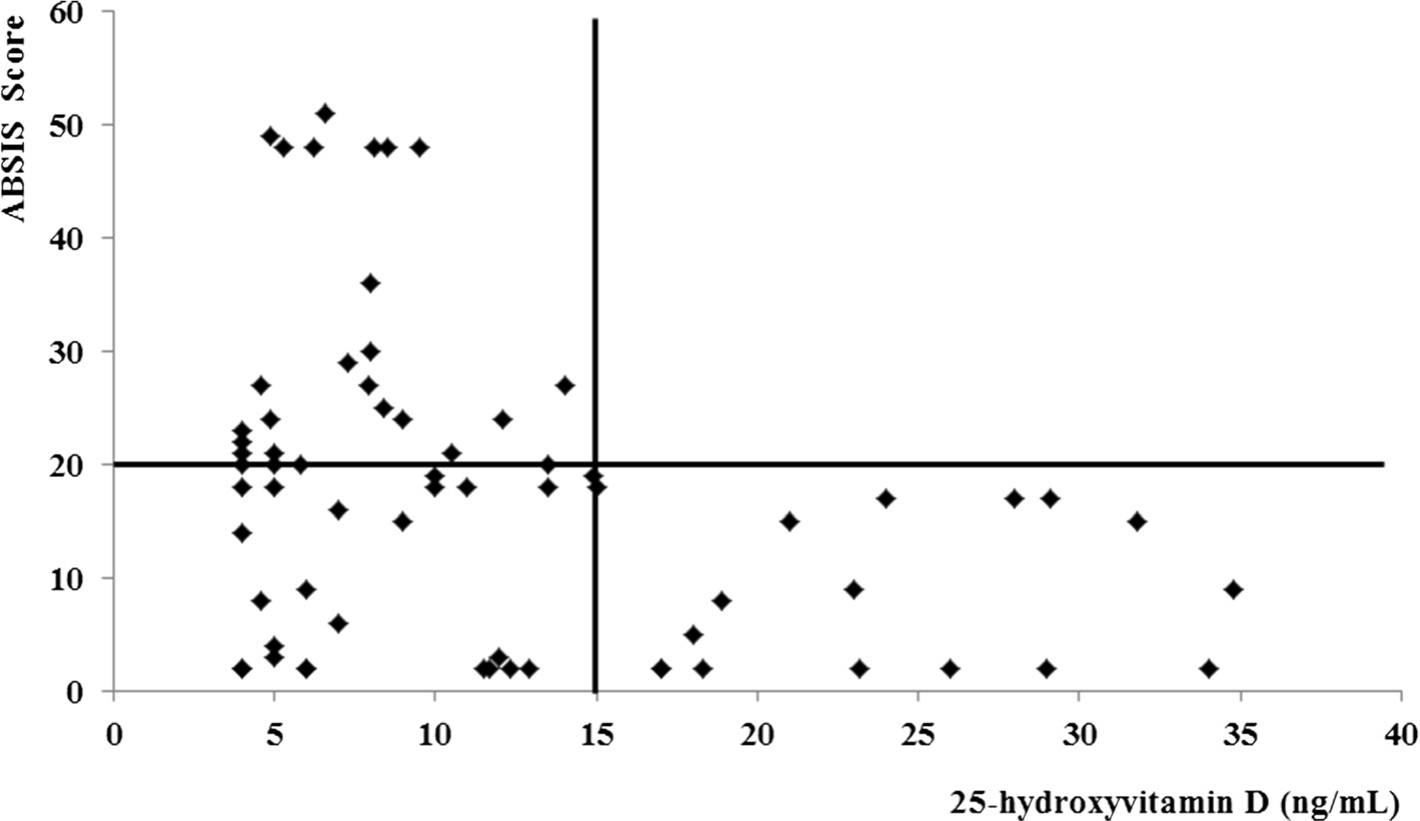
**Correlation between vitamin D levels and ABSIS score.** ABSIS score: bullous skin disorder intensity score (see reference #26). In the whole group of patients the ABSIS score was inversely associated with 25OHVitD levels (R = −0.36, p < 0.005). All patients with 25OHVitD levels above 15 ng/ml showed ABSIS score <20. On the other hand, in the presence of 25OHVitD levels below 15 ng/ml 22 and 28 patients showed ABSIS score <20 and ≥20 respectively.

In the whole group of patients, the 25OHVitD levels were inversely associated with ABSIS score (R = −0.36, p < 0.005, Figure 1) and with age (R = −0.34, p = 0.05). Separately analyzing PV and BP patients, this association remained significant only in the latter group (R = −0.15, p = 0.4 and R = −0.39, p = 0.03, respectively).

The multivariate regression analysis showed that the ABSIS score was associated with 25OHVitD levels (β = −3.2, P = 0.009), regardless of age (β = 0.11, P = 0.337). Interestingly, all patients with 25OHVitD levels above 15 ng/ml showed ABSIS score <20. On the other hand, in the presence of 25OHVitD levels below 15 ng/ml 22 and 28 patients showed ABSIS score <20 and ≥20, respectively.

## Discussion

This study was aimed to investigate the issue of hypovitaminosis D and skeletal health in patients with autoimmune bullous skin diseases. We found that PV and BP patients with active disease had lower 25OHVitD levels and higher prevalence of severe hypovitaminosis D as compared with matched control subjects. Moreover, PV and BP patients showed increased prevalence of vertebral fractures in spite of not reduced BMD. Finally, the disease severity was found to be associated with 25OHVitD levels regardless of age.

Previous studies gave discordant results regarding the prevalence of hypovitaminosis D in patients with autoimmune bullous skin diseases. Indeed, our previous finding of low 25OHVitD levels in PV and BP patients [15] was confirmed by subsequent data [16] and by the results of the present extension study, but not by two other studies [17,18]. This discordances may be due to the small sample size of the previous studies and to the fact that hypovitaminosis D is highly prevalent even in the normal population [19], as also evidenced by the mean 25OHVitD levels within the range of insufficiency in our control group. Pooling data from our previous and present extension study together consented us to obtain the largest sample of PV and BP patients investigated so far. Our data clearly demonstrated that vitamin D levels are reduced in both PV and BP patients, suggesting that hypovitaminosis D may have a pathogenetic role in autoimmune bullous diseases. It is well-known that PV and BP are autoantibody-driven autoimmune disorders in which both T cells and B cells with auto-reactivity towards DSG3 and BP180/BP230, respectively, are necessary for their pathogenesis [10]. During acute phase of PV and BP, auto-reactive T helper (Th)1 and Th2 lymphocytes cooperatively play a role in the development of the disease process [26]. Recently, a focus has been placed on the contribution of the newly discovered Th17 subset to the pathophysiology of both diseases, since increased serum levels of the Th17-derived interleukin (IL)-17, a pro-inflammatory cytokine contributing to tissue damage, have been found in autoimmune blistering diseases [17]. Finally, an impaired function of T regulatory cells (T regs), whose immune-surveillance action is critical in preventing autoimmunity, has been observed during the active phase of both diseases [17]. Thus, considering that vitamin D has modulatory effects on B lymphocyte proliferation and immunoglobulin synthesis [20; 27], its deficiency may increase autoantibody production in autoimmune bullous diseases. Moreover, vitamin D inhibits expression of Th1 cytokines, such as IL-2, interferon-γ and tumor necrosis factor-α, thus providing protection against autoimmunity [20]. In, contrast, the effects of vitamin D on Th2 cells are conflicting, since it seems to inhibit Th2 cell differentiation, but enhances the secretion of Th2-derived cytokines, like IL-4 and IL-5 [20,16]. Finally, vitamin D can induce an increase in T regs and a decrease in expression of IL-17 [20; 27], dampening autoimmune response and inflammation. Thus, vitamin D supplementation may improve autoimmune disorders associated with its deficiency like PV and BP. Given the immune-modulatory properties of vitamin D [13,14,20,27,28], our finding of the inverse correlation between 25OHVitD levels and disease intensity, as evaluated by the ABSIS score, further supports that the low vitamin D levels, and most notably severe hypovitaminosis D, may play a role in exacerbating the autoimmune response in bullous diseases. In keeping, we found that the disease intensity is invariably low in the presence of 25OHVitD levels above 15 ng/ml, while it is highly variable in the presence of severe hypovitaminosis D (Figure 1).

The possible association between the degree of the autoimmune response and vitamin D is also suggested by the presence in BP patients of an inverse correlation between 25OHVitD and the BP180 serum levels. In PV patients, the lack of a similar association between 25OHVitD levels and Dsg3-specific autoantibodies or Dsg1-specific autoantibodies levels can be due to the dramatically low 25OHVitD levels in most patients of the study that may have hidden this relationship.

Beside these possible pathophysiological aspects, the present study provides also some noteworthy clinical information. Indeed, previous studies reported that osteoporosis was more prevalent in PV patients as compared with controls regardless of the use of glucocorticoids [29] and that the glucocorticoid-induced BMD decrease may be prevented by alendronate [30]. In the present study, however, the finding of increased risk of fractures in PV and BP patients regardless of BMD, suggests the need of searching for morphometric VFx in these patients, even in the absence of clearly reduced BMD, as the risk of fractures seems to be not entirely explained by a decrease in bone density. It is possible to hypothesize that in autoimmune bullous skin diseases a reduction of bone quality contributes to the increased prevalence of fractures, similarly to what happens in other forms of secondary osteoporosis [31]. In keeping, ALP and calcium levels were found to be reduced in our both PV and BP patients, likely indicating a low bone turnover and in particular impaired osteoblastic activity [32], which are known to be important contributors for the maintenance of bone quality. From a clinical point of view, the awareness of a bone involvement before the initiation of the commonly used steroid therapy, may consent to prevent the occurrence of subsequent clinical vertebral or hip fractures, which represent a medical emergency, and may worsen even further the general health in patients affected with these rare but potentially life-threatening skin diseases. As a consequence, the prophylaxis of bone fractures in patients with autoimmune bullous diseases should be considered even in premenopausal women and men regardless of the use of steroids.

Finally, the finding that the disease intensity is invariably low in the presence of 25OHVitD levels above 15 ng/ml (Figure 1) is completely novel. Although entirely speculative, it is possible to hypothesize that 25OHVitD levels within the normal range or at least above 15 ng/ml are needed to reduce the risk of the recrudescence and/or relapses of PV and BP. Further longitudinal and intervention studies are needed to confirm this hypothesis.

In summary, as compared with the previous study [15], the presents extension study provides some novel important findings. Firstly, it demonstrates the presence of an increased prevalence of VFx not only in BP but also in PV patients separately analyzed. Secondly, the increased number of patients included in the extension study allowed to clarify that BMD is of limited usefulness in evaluating the propensity to VFx in patients with autoimmune bullous skin diseases. Finally, the extension study consented to reliably investigate the association between the 25OHVitD levels and disease intensity, suggesting that the severely reduced vitamin D levels (particularly below 15 ng/mL) may play a role in exacerbating the autoimmune response in these patients.

The present study has some limits. Firstly, its cross-sectional and observational design does not allow to look for causality. Secondly, we did not assess the cytokine milieu and the most sensitive markers of bone apposition and resorption that could have been more informative. Finally, the association between 25OHVitD levels with other routinely used clinical scores, such as pemphigus disease area index (PDAI) and bullous pemphigoid disease area index (BPDAI) [24], has been not assessed. However, the study has several strengths. Firstly, we could investigate a relatively large sample of patients (considering the rarity of these diseases) and obtain a quite comprehensive picture of the skeletal involvement in PV and BP. Secondly, we studied only naïve patients and compared them with strictly matched controls (who had the same setting of inpatients). Finally, we took into account some potentially confounding variables (i.e. smoking and sun exposure) that might had influenced the results of previous studies.

## Conclusions

The present study shows that PV and BP patients with active disease have increased prevalence of severe hypovitaminosis D, reduced 25OHVitD levels, inversely associated with disease intensity, and increased prevalence of vertebral fractures in spite of not reduced BMD. Of interest, the association between the ABSIS score of cutaneous involvement and the vitamin D level, which has never been previously reported by other investigators, may have a direct implication in clinical practice. All the above findings support the hypothesis of an influence of vitamin D in triggering or exacerbating these autoimmune disorders and suggest the need of evaluating the skeletal health in PV and BP patients regardless of steroid therapy. Further studies are needed to investigate the possible causative role of hypovitaminosis D in PV and BP and the possible therapeutic implications.

## Additional file

Additional file 1: Table S1. Clinical characteristics of patients and controls included in the extension study. **Table S2.** Clinical characteristics of the whole sample of patients and controls.
