# Supplementary material for: Vitamin D and skeletal health in autoimmune bullous skin diseases: a case control study
Source: Orphanet J Rare Dis. 2015 Feb 3;10:8. doi: 10.1186/s13023-015-0230-0 (PMC4323030; doi:10.1186/s13023-015-0230-0)
Supplement: Additional file 1: Table S1. — Clinical characteristics of patients and controls included in the extension study. Table S2. Clinical characteristics of the whole sample of patients and controls. [file 13023_2015_230_MOESM1_ESM.docx]

**Additional file 1: Table S1** Clinical characteristics of patients and controls included in the extension study

|  | **Controls**  **(n=39)** | **Patients**  **(n=39)** | **P value** |
| --- | --- | --- | --- |
| **Age** (years) | 64.1 ± 20.9  (27 – 90) | 67.2 ± 17.1  (27 – 90) | 0.477 |
| **Gender** (females) | 21  (53.8) | 21  (53.8) | 1.000 |
| **BMI** (kg/m^2^) | 26.7 ± 4.4  (19.9 – 37.0) | 25.2 ± 4.3  (18.4 – 36.7) | 0.123 |
| **N. of current smokers** (%) | 10  (25.6) | 6  (15.4) | 0.262 |
| **N. of subjects with history of clinical fractures** (%) | 4  (10.3) | 2  (5.1) | 0.395 |
| **Sun Exposure Score** | 11.5 ± 8.5  (2 – 28) | 11.5 ± 6.8  (2 – 33) | 0.977 |
| **Calcium** (mg/dL) | 9.25 ± 0.29  (8.6 – 10.0) | 9.11 ± 0.41  (8.2 – 10.0) | 0.07 |
| **25(OH)Vitamin D** (ng/mL) | 22.2 ± 10.8  (6.4 – 42) | 12.7 ± 9.5  (4.0 – 34.8) | <0.0001 |
| **N. of subjects with severe hypovitaminosis D** (%) | 11  (28.2) | 22  (56.4) | 0.012 |
| **Alkaline Phosphatase** (U/L) | 69.8 ± 14.9  (39 – 115) | 60.3 ± 18.1  (34 – 128) | 0.14 |
| **LS BMD** (Z-score) | 0.08 ± 1.49  (-2.6 – 3.7) | 0.29 ± 1.74  (-3.2 – 3.3) | 0.568 |
| **FN BMD** (Z-score) | -0.29 ± 1.01  (-2.0 – 2.7) | -0.06 ± 1.22  (-2.9 – 3.0) | 0.362 |
| **N. of subjects with T-score < -2.5 at any site** (%) | 7  (17.9) | 13  (33.3) | 0.120 |
| **N. of subjects with vertebral fractures** (%) | 12  (30.8) | 23  (59.0) | 0.012 |

Data are mean±SD with range or percentage in parentheses for continuous or categorical variable, respectively. Severe hypovitaminosis D was defined in the presence of 25OHVitD levels <12 ng/mL (reference interval: 30-120 ng/ml). Sun Exposure score: participants’ recollection of daily sun exposure over the previous week was assessed via a questionnaire administered (see reference #30). Z-score, T-score: difference in standard deviation units in relation to the reference healthy population of same age (Z-score) and of the young adults (T-score).

Alkaline Phosphatase reference interval: 35-104 U/L. BMI: body mass index; BMD: bone mineral density; LS: lumbar spine; FT: total femur; FN: femoral neck.

**Table S2** Clinical characteristics of the whole sample of patients and controls

|  | **Controls**  **(n=67)** | **Patients**  **(n=67)** | **P value** |
| --- | --- | --- | --- |
| **Age** (years) | 64.7 ± 16.9  (27 – 90) | 66.8 ± 17.0  (27 – 90) | 0.486 |
| **Gender** (females) | 36  (53.7) | 36  (53.7) | 1.000 |
| **BMI** (kg/m^2^) | 26.9 ± 4.5  (19.9 – 37.0) | 25.9 ± 4.6  (17.6 – 39.0) | 0.185 |
| **N. of current smokers** (%) | 15  (22.4) | 10  (14.9) | 0.268 |
| **N. of subjects with history of clinical fractures** (%) | 9  (13.4) | 4  (6.0) | 0.242 |
| **Sun Exposure Score** | 11.8 ± 7.3  (2 – 28) | 12.1 ± 6.6  (2 – 33) | 0.84 |
| **Calcium** (mg/dL) | 9.27 ± 0.27  (8.6 – 10.0) | 9.05 ± 0.37  (8.2 – 10.0) | <0.0001 |
| **25(OH)Vitamin D** (ng/mL) | 22.3 ± 12.9  (4.0 – 79.0) | 11.8 ± 8.3  (4.0 – 34.8) | <0.0001 |
| **N. of subjects with severe hypovitaminosis D** (%) | 18  (26.9) | 41  (61.2) | 0.000 |
| **Alkaline Phosphatase** (U/L) | 71.7 ± 17.9  (38 – 115) | 62.5 ± 18.9  (34 – 128) | 0.006 |
| **LS BMD** (Z-score) | 0.10 ± 1.46  (-2.6 – 3.8) | 0.21 ± 1.67  (-3.2 – 3.3) | 0.660 |
| **FN BMD** (Z-score) | -0.19 ± 1.02  (-2.0 – 2.7) | -0.09 ± 1.10  (-2.9 – 3.0) | 0.586 |
| **N. of subjects with T-score < -2.5 at any site** (%) | 13  (19.4) | 20  (29.9) | 0.160 |
| **N. of subjects with vertebral fractures** (%) | 21  (31.3) | 40  (59.7) | 0.001 |

Data are mean±SD with range or percentage in parentheses for continuous or categorical variable, respectively. Severe hypovitaminosis D was defined in the presence of 25OHVitD levels <12 ng/mL (reference interval: 30-120 ng/ml).

Sun Exposure score: participants’ recollection of daily sun exposure over the previous week was assessed via a questionnaire administered (see reference #30)

Z-score, T-score: difference in standard deviation units in relation to the reference healthy population of same age (Z-score) and of the young adults (T-score).

Alkaline Phosphatase reference interval: 35-104 U/L. BMI: body mass index; BMD: bone mineral density; LS: lumbar spine; FT: total femur; FN: femoral neck.
